# Canine REIC/Dkk-3 interacts with SGTA and restores androgen receptor signalling in androgen-independent prostate cancer cell lines

**DOI:** 10.1186/s12917-017-1094-4

**Published:** 2017-06-09

**Authors:** Yuiko Kato, Kazuhiko Ochiai, Shota Kawakami, Nobuhiro Nakao, Daigo Azakami, Makoto Bonkobara, Masaki Michishita, Masami Morimatsu, Masami Watanabe, Toshinori Omi

**Affiliations:** 1 School of Veterinary Nursing and Technology, Faculty of Veterinary Science, Nippon Veterinary and Life Science University, Tokyo, 180-8602 Japan; 2 Laboratory of Animal Physiology, School of Animal Science, Nippon Veterinary and Life Science University, Tokyo, 180-8602 Japan; 3 Department of Veterinary Clinical Pathology, Faculty of Veterinary Science, Nippon Veterinary and Life Science University, Tokyo, 180-8602 Japan; 4 Department of Veterinary Pathology, Faculty of Veterinary Science, Nippon Veterinary and Life Science University, Tokyo, 180-8602 Japan; 5 Laboratory of Laboratory Animal Science and Medicine, Department of Disease Control, Graduate School of Veterinary Medicine, Hokkaido University, Sapporo, 060-0818 Japan; 6 Department of Urology, Graduate School of Medicine, Dentistry and Pharmaceutical Sciences, Okayama University, Okayama, 700-8558 Japan

**Keywords:** Androgen receptor (AR) signalling, Canine, Prostate cancer, Reduced expression in immortalised cells (REIC/Dkk-3), Small glutamine-rich tetratricopeptide repeat-containing protein α (SGTA)

## Abstract

**Background:**

The pathological condition of canine prostate cancer resembles that of human androgen-independent prostate cancer. Both canine and human androgen receptor (AR) signalling are inhibited by overexpression of the dimerized co-chaperone small glutamine-rich tetratricopeptide repeat-containing protein α (SGTA), which is considered to cause the development of androgen-independency. Reduced expression in immortalised cells (REIC/Dkk-3) interferes with SGTA dimerization and rescues AR signalling. This study aimed to assess the effects of REIC/Dkk-3 and SGTA interactions on AR signalling in the canine androgen-independent prostate cancer cell line CHP-1.

**Results:**

Mammalian two-hybrid and Halo-tagged pull-down assays showed that canine REIC/Dkk-3 interacted with SGTA and interfered with SGTA dimerization. Additionally, reporter assays revealed that canine REIC/Dkk-3 restored AR signalling in both human and canine androgen-independent prostate cancer cells. Therefore, we confirmed the interaction between canine SGTA and REIC/Dkk-3, as well as their role in AR signalling.

**Conclusions:**

Our results suggest that this interaction might contribute to the development of a novel strategy for androgen-independent prostate cancer treatment. Moreover, we established the canine androgen-independent prostate cancer model as a suitable animal model for the study of this type of treatment-refractory human cancer.

**Electronic supplementary material:**

The online version of this article (doi:10.1186/s12917-017-1094-4) contains supplementary material, which is available to authorized users.

## Background

Prostate cancer is the most common human male cancer in Western society and is currently rising in Eastern Asia [1]. Prostate cancer accounts for about 25% of all cancers in the male population of the United States [2]. For its treatment, hormonal strategies [e.g., androgen ablation therapy and/or androgen receptor (AR) antagonists] are used to prevent AR signalling associated with the development and progression of prostate cancer. Although the majority of prostate tumours respond to androgen ablation, a proportion of the cases develop as androgen-independent pathological conditions [3]. Canine prostate cancer shows similar characteristics to human androgen-independent prostate cancer, which progresses without androgen stimulation and is refractory to anti-androgen therapy [4]. To study the similarities between the pathological conditions of both canine and human androgen-independent prostate cancer, we have established a canine androgen-independent prostate cancer cell line, CHP-1 [5].

Reduced expression in immortalised cells (*REIC*), initially discovered as a tumour suppressor gene, is identical to the Dickkopf-3 (*Dkk-3*) gene, which is a member of the Dickkopf gene family [6]. *REIC/Dkk-3* is ubiquitously expressed in normal cells, whereas its expression is significantly downregulated in various cancer cell types, including human and canine prostate cancer [7]. Downregulation of *REIC/Dkk-3* is associated with the malignancy of various cancer types [7–9]. Previous studies showed that overexpression of *REIC/Dkk-3* using adenoviral or plasmid vectors induces apoptosis in various cancer cell lines, but not in normal cells, via c-Jun-NH2-terminal kinase (JNK) and c-Jun activation, and endoplasmic reticulum (ER) stress signalling [7, 10–13].

Small glutamine-rich tetratricopeptide repeat-containing protein α (SGTA) is an Hsp70/Hsp90-associated co-chaperone that interacts with the hinge region of the human AR and interferes with its cytoplasmic maturation [3]. The dimerized form of SGTA was identified as a negative regulator of AR transport to the nucleus by hampering the interaction between the cytoplasmic AR complex and the dynein motor complex [14–18]. Our recent study revealed that the overexpression of *REIC/Dkk-3* in the human androgen-independent prostate cancer cell line PC3 upregulates AR signalling and restores the expression of prostate specific antigen (PSA) by interfering with the dimerization of SGTA [19].

We have previously cloned canine SGTA and showed that its expression levels were significantly higher in canine prostate cancer tissues compared to that in prostate hyperplasia [20]. In addition, we showed that canine SGTA played an inhibitory role in AR signalling both in canine and human androgen-independent prostate cancer cells [5, 20]. From these findings, we were able to hypothesize that canine SGTA leads to an androgen-independent condition in canine prostate cancer. Our aim was to investigate whether the interaction between canine REIC/Dkk-3 and SGTA affects AR signalling and might contribute to the development of new strategies in canine androgen-independent prostate cancer treatment. In this study, we describe the interaction between canine REIC/Dkk-3 and SGTA, and the role of canine REIC/Dkk-3 in AR signalling recovery, previously inhibited by SGTA.

## Methods

### Cell lines

The human 293 T and PC3 cell lines were provided by the American Type Culture Collection (ATCC, Rockville, MD). The canine prostate cancer CHP-1 cell line was established from a prostate mass collected immediately following surgery of a tumour-bearing, 10-year-old, castrated male Jack Russell Terrier breed dog in our university [5]. The 293 T and the CHP-1 cells were maintained in Dulbecco’s modified Eagle’s medium (DMEM) (Wako, Osaka, Japan) and PC3 cells were maintained in Ham’s F12 medium (Wako) supplemented with 10% foetal bovine serum (FBS), penicillin (50 IU/mL), and streptomycin (50 μg/mL) under a humidified atmosphere with 5% CO_2_ at 37 °C.

### Mammalian two-hybrid (MTH) assay

For the MTH assay, the full-length open reading frame (ORF) of canine SGTA and REIC/Dkk-3 cDNA was cloned between the *EcoR*I and *Mlu*I sites of a pM GAL4 DNA-binding domain cloning plasmid (GAL4-DBD) and pVP16 transactivation domain cloning plasmid (VP16-AD) (Clontech Laboratories, Palo Alto, CA, USA), respectively. In addition, the full-length ORF of canine REIC/Dkk-3 cDNA was cloned between the *Xho*I and *EcoR*I sites of the pMACS KK HA(C) vector cloning plasmid (Miltenyi Biotec, Bergisch Gladbach, Germany). Primers were used in this study was described in Additional file 1: Table S1. Approximately 2 × 10^5^ 293 T cells per well in 24-well plates were co-transfected with 100 ng of pM, pVP16, pMACS KK HA(C), and pFR-Luc firefly luciferase reporter plasmid (Promega, Madison, WI, USA), and 0.2 ng of phRL-TK *Renilla* luciferase reporter plasmid (Promega). The cells were harvested 48 h after transfection, and the luciferase activity was measured using the dual-luciferase reporter assay system (Promega). The luciferase activity was normalized to the value of the *Renilla* luciferase activity [21].

### Pull-down (PD) assay

A Halo-tagged canine SGTA was cloned into a pFN21A vector (Promega). To generate haemagglutinin (HA)-tag fusion proteins, the *Xho*I/*EcoR*I fragment of canine REIC/Dkk-3 cDNA was cloned into a pMACS KK HA(C) vector. The expression of Halo and HA-tagged constructs was induced in 293 T cells using the FuGENE® HD transfection reagent (Promega), and the transfected cells were grown for 48 h. Cells were harvested by centrifugation and washed with Phosphate-buffered Saline (PBS). The cells were lysed in Mammalian Lysis Buffer (Promega) with a Protease Inhibitor Cocktail (Promega) for 15 min, and the cellular debris was cleared by centrifugation. To the supernatant, 100 μL of HaloLink™ Resin (Promega), equilibrated with TBS including 0.05% IGEPAL CA-630 (TBS+), were added. The samples were incubated for 20 min at 25 °C with rotation. The supernatant was discarded; the resin was washed three times with TBS+ and resuspended in sodium dodecyl sulphate-polyacrylamide gel electrophoresis (SDS-PAGE) loading buffer. The samples were analysed by western blot analysis using an anti-Halo antibody (1:2000) (G9281, Promega), an anti-haemagglutinin (HA) tag antibody (1:2000) (MBL-561, Medical and Biological Laboratories (MBL), Aichi, Japan) (1:2000), and a horseradish peroxidase-conjugated anti-rabbit IgG antibody (GE Healthcare, Waukesha, WI, USA). Blots were developed using the EzWestLumi plus reagent (ATTO, Tokyo, Japan).

### Immunostaining

Immunocytochemical co-staining for forced-expressed Halo-tagged canine REIC/Dkk-3 and endogenous SGTA in CHP-1 cells was performed using the mouse monoclonal anti-Halo antibody (1:100) (G9211, Promega) and rabbit polyclonal anti-SGTA antibody (1:100) (sc-292,025, Santa Cruz Biotechnology, Santa Cruz, CA, USA). Cells were plated and cultured to 30–40% confluence in Lab-Tek chambers (Nalgene, Rochester, NY, USA), and were transfected with the pFN21A vector containing Halo-tagged canine REIC/Dkk-3 by FuGENE HD (Promega). Forty-eight hours after transfection, the cells were fixed in 4% paraformaldehyde in 100 mM phosphate buffer and blocked with 5% normal goat serum in PBS. After the samples were incubated overnight at 4 °C with primary antibodies, they were incubated for 1 h at 25 °C with Alexa Fluor 488 anti-mouse and Alexa Fluor 594 anti-rabbit secondary antibodies (Invitrogen, Carlsbad, CA, USA). To stain the nuclei, the cells were incubated with Hoechst 33,342 (Dojindo Laboratories, Kumamoto, Japan) for 15 min at room temperature. Fluorescent staining was visualised and analysed under a fluorescence microscope system equipped with an analytical software program (BZ-9000, Keyence, Osaka, Japan).

### Transactivation assays

For this experiment, 10% charcoal-stripped FBS was used for the cell culture. Both PC3 and CHP-1 prostate cancer cells (2 × 10^5^/well in 24-well plates, 500 μL medium/well) were transfected with 100 ng of pEGFP C1 AR vector containing the full-length AR (Plasmid ID: 28,235, Addgene, Cambridge, MA, USA) [22], 100 ng of p159-pPr-luc vector containing a firefly luciferase reporter gene downstream of the rat probasin gene promoter (Plasmid ID: 8392, Addgene) [23], 100 ng of pFN21A-SGTA, 200 ng of pMACS KK HA(C)-REIC/Dkk-3, and 25 ng of phRL-tk *Renilla* luciferase reporter plasmid by using the Lipofectamine 2000 reagent (Thermo Fisher Scientific, Waltham, MA, USA). After 48 h of transfection, the cells were treated for 24 h with control vehicle (ethanol) or dihydrotestosterone (DHT) (Sigma, St Louis, MO, USA), and were assayed for luciferase activity using a Dual-Luciferase Reporter Assay System (Promega). All transfection mixes were balanced with the appropriate empty vectors in terms of the ratio of the expression vectors and total plasmids.

### Immunoblotting

Total protein was extracted from the cells using Mammalian Lysis Buffer (Promega) with Protease Inhibitor Cocktail (Promega) for 15 min, and the cellular debris was cleared by centrifugation. Canine control fibroblasts were collected from normal breast tissue of an 8-year-old female Chihuahua undergoing contraceptive treatment. Western blot analysis was performed as previously described [24]. Approximately 10 μg of extracted protein were analysed with the specific primary antibodies as follows: rabbit polyclonal anti-EGFP (MBL-598, MBL), rabbit polyclonal anti-SGTA (sc-292,025), rabbit polyclonal anti-REIC/Dkk-3 (10365–1-AP, Proteintech, Chicago, IL, USA), and anti-β-actin (sc-69,879, Santa Cruz Biotechnology).

### Statistical analysis

The data are shown as the mean ± SE. Student’s *t*-test or one-way analysis of variance (ANOVA) followed by Bonferroni test were performed to assess the significance of differences between two or more groups, respectively. Differences were considered significant with *P* < 0.05.

## Results

### REIC/Dkk-3 expression in canine prostate cancer cell lines and its interaction with SGTA

**Fig. 1:**
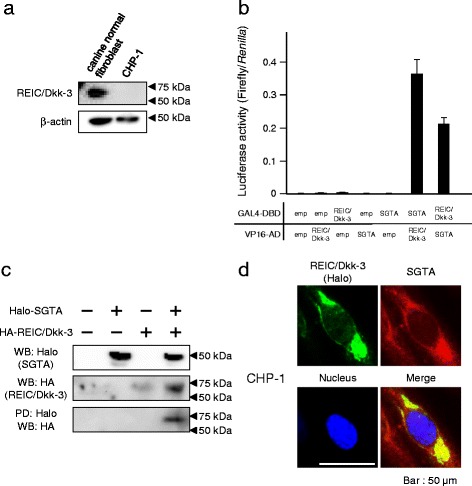
Interaction between canine SGTA and REIC/Dkk-3. **a** Western blot analysis showing the expression profile of canine REIC/Dkk-3 and control β-actin in canine control fibroblasts and androgen-independent cell line CHP-1. **b** The interaction between canine SGTA and REIC/Dkk-3 was demonstrated by a mammalian two-hybrid (MTH) assay. DBD: GAL4-DNA-binding domain fusion protein. AD: VP16 transactivation domain fusion protein. The results were obtained from three independent experiments. **c** Western blot and pull-down assay results obtained from haemagglutinin (HA)-tagged REIC/Dkk-3 and Halo-tagged SGTA transfected 293 T cells lysates. **d** Co-localization of canine forced-expressed Halo-tagged REIC/Dkk-3 and endogenous SGTA was examined by double-immunofluorescence staining and observed by fluorescence microscopy. The images in green and red show the intracellular localization of Halo-tagged REIC/Dkk-3 and SGTA, respectively. The areas of overlap between REIC/Dkk-3 and SGTA are shown in yellow in the merged image

Canine REIC/Dkk-3 proteins were not expressed in canine androgen-independent prostate cancer CHP-1 cells as confirmed by western blot analysis, compared with canine control fibroblasts (Fig. 1a). Further, we analysed the interaction between canine REIC/Dkk-3 and SGTA by an MTH assay in 293 T cells using co-transfection and expression of each protein cloned into GAL4-DBD and VP16-AD plasmids, respectively. The luciferase activity, which reflects the binding intensity of each fusion protein, was increased in comparison to the empty vectors used as negative controls (Fig. 1b). The PD assay, performed to confirm the interaction between canine SGTA and REIC/Dkk-3, showed that the canine fused HA-REIC/Dkk-3 was detected in the canine Halo-tagged SGTA co-expression condition (Fig. 1c). Therefore, the interaction between canine SGTA and REIC/Dkk-3 was demonstrated by both, the MTH and PD assays. To further verify the interaction between canine REIC/Dkk-3 and SGTA in canine cells, immunocytochemistry was performed on CHP-1 cells that forced-expressed Halo-tagged canine REID/Dkk-3 using anti-Halo and anti-SGTA antibodies. Immunostaining revealed that Halo-tagged REIC/Dkk-3 and SGTA showed cytoplasmic localization with a similar distribution pattern, suggesting that their interaction and functions occur in the same locations (Fig. 1d).

### Canine REIC/Dkk-3 interference with SGTA dimerization

**Fig. 2:**
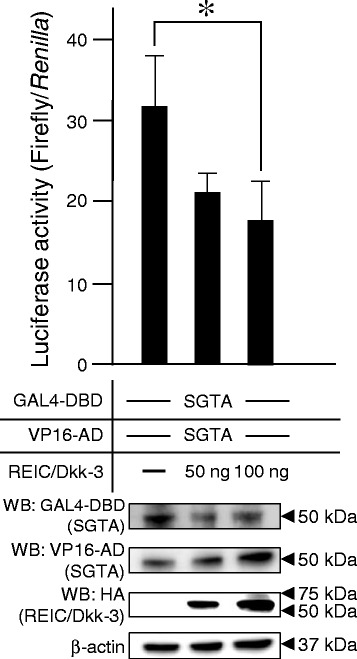
Canine REIC/Dkk-3 interference in SGTA dimerization. The upper graph shows the role of canine REIC/Dkk-3, which interferes with SGTA dimerization, examined by a modified MTH assay. 293 T cells were co-transfected with DBD- or AD-fused canine SGTA plasmids and haemagglutinin (HA)-tagged REIC/Dkk-3. Luciferase activity in cell lysates was determined 48 h after transfection. The results were obtained from three independent experiments. The results show a significant difference between the SGTA alone and with the addition of 100 ng of canine REIC/Dkk-3 (*: *P* < 0.05, ANOVA). The lower panel depicts the western blot analysis showing the expression of DBD- and AD- fused canine SGTA, and haemagglutinin (HA)-tagged REIC/Dkk-3 after co-transfection in a parallel experiment with that depicted in the upper graph

We evaluated the interference of canine REIC/Dkk-3 in SGTA dimerization by using a modified-MTH assay, which has been employed in our previous studies [19, 25]. Canine REIC/Dkk-3 was co-transfected with canine SGTA, cloned in GAL4-DBD and VP16-AD plasmids, respectively. The luciferase activity was significantly decreased in the 100-ng canine REIC/Dkk-3 condition compared to the expression of canine SGTA alone (Fig. 2).

### Interaction between canine REIC/Dkk-3 and SGTA regulates AR signalling activity

**Fig. 3:**
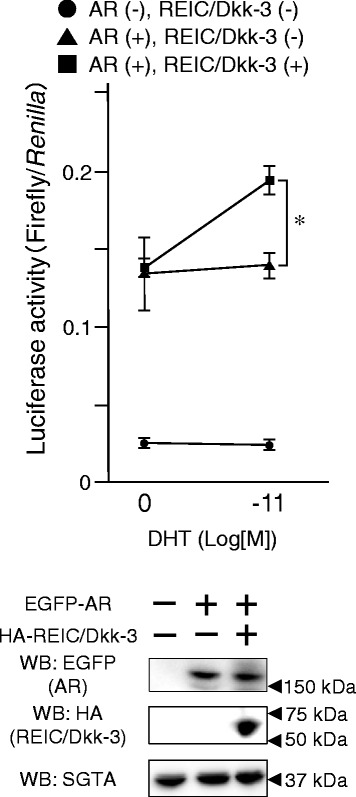
Interaction between canine SGTA and REIC/Dkk-3 regulates AR signalling in canine androgen-independent prostate cancer cell line CHP-1. The upper graph shows the effects of canine REIC/Dkk-3 expression on AR signalling in the AR absent or present conditions with/without DHT stimulation in CHP-1 cells. Cells were co-transfected with the p159-pPr-Luc plasmid and treated with dihydrotestosterone (DHT). The value for the baseline was obtained using the control vehicle. The results were obtained from three independent experiments, and show a significant difference in AR activity based on REIC/Dkk-3 expression (*: *P* < 0.05, ANOVA). The lower figure shows the western blot results for the expression of EGFP-tagged AR, HA-tagged REIC/Dkk-3 and endogenous SGTA in CHP-1 cells after co-transfection in a parallel experiment with that depicted in the upper graph

To evaluate the interaction between canine REIC/Dkk-3 and SGTA in the AR signal transduction pathway in canine prostate cancer, we first measured the extent of AR signalling activation by the probasin promoter-driven luciferase reporter assay [3, 19]. We expressed the AR and/or canine REIC/Dkk-3 in canine androgen-independent prostate cancer CHP-1 cells. The expression of canine REIC/Dkk-3 induced a significant increase in AR signalling with DHT stimulation (Fig. 3). This result suggests that REIC/Dkk-3 upregulates AR signalling in canine androgen-independent prostate cancer.

**Fig. 4:**
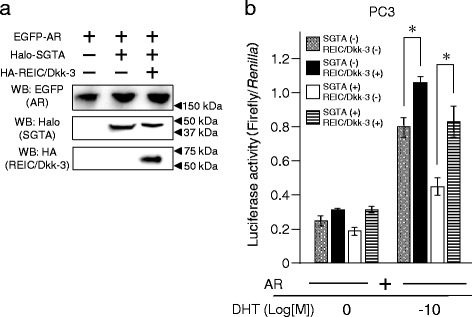
Co-transfection with exogenous canine SGTA and REIC/Dkk-3 regulates AR signalling in human androgen-independent cancer cell line PC3. **a** Western blot analysis showing the expression of EGFP-tagged AR, Halo-tagged canine SGTA and HA-tagged canine REIC/Dkk-3 after co-transfection in a parallel experiment with that depicted in the graph **b**. **b** The effects of canine SGTA expression on the AR activity were examined in human prostate cancer PC3 cells under co-transfection with the AR, canine SGTA, and canine REIC/Dkk-3, with/without DHT. The results were obtained from three independent experiments, and show a significant difference in AR activity based on SGTA and/or REIC/Dkk-3 expression (*: *P* < 0.05, Student’s *t*-test). All transfection mixes were balanced with the appropriate empty vectors in terms of the ratio of the expression vectors and total plasmids. The value for the baseline was obtained using the control vehicle

To investigate this mechanism, we repeated this assay in human androgen-independent prostate cancer PC3 cells. We expressed canine HA-REIC/Dkk-3 and/or Halo-SGTA together with the AR in PC3 cells (Fig. 4a). Canine REIC/Dkk-3 significantly increased, whereas canine SGTA decreased basal AR signalling and the co-expression of canine REIC/Dkk-3 recovered the signalling suppressed by SGTA in DHT-stimulated conditions (Fig. 4b). These results suggest that AR signalling is regulated by the canine REIC/Dkk-3 tumour suppressor, which interacts and interferes with SGTA functions and suppresses AR signalling in canine and human androgen-independent prostate cancer cell lines.

## Discussion

Here, we demonstrated that the canine tumour suppressor REIC/Dkk-3 interacts with SGTA, which is a co-chaperone known to interact with Hsp70/Hsp90 and the cytosolic immature AR complex [3, 17, 19, 26]. Dimerized SGTA is an inhibitor of dynein motor-dependent AR transport and signalling, which is the cause of the androgen-independent condition in human prostate cancer [3, 17]. Canine SGTA also forms dimers and suppresses AR signal transduction [5, 20]. REIC/Dkk-3 interacts with SGTA and interferes with its dimerization recovering AR translocation and signalling [19]. The interaction between REIC/Dkk-3 and SGTA shown in this study suggests that REIC/Dkk-3 may be a crucial step in the AR signalling pathway in dogs (Fig. 1b and c). Furthermore, overexpressed canine REIC/Dkk-3 interferes with SGTA dimerization and has the potential to function as an upregulator in the AR signalling pathway (Fig. 2). The underexpression of REIC/Dkk-3 in CHP-1 (Fig. 1a) suggests that the lack of this protein causes the androgen-independency in canine prostate cancer. In dogs, nearly all prostate cancers are androgen-independent, and thus, we expected that prostate cancer cell lines derived from dog e.g. DPC-1 [27], ACe-1 [28], and Leo [29] may downregulate the expression of REIC/Dkk-3.

In addition, we demonstrated that the overexpression of the canine REIC/Dkk-3 protein upregulates AR signal transduction in both canine and human androgen-independent prostate cancer cell lines. This result indicates that REIC/Dkk-3 activates AR transport by binding to SGTA and interfering with its dimerization and negative regulation (Figs. 3 and 4b). In humans, adenovirus-mediated REIC/Dkk-3 overexpression in various cancers, including prostate cancer, induces tumour specific apoptosis [7–9]. Furthermore, a canine study has shown an apoptotic function of REIC/Dkk-3 in cell lines established from a canine mammary gland tumour [30]. These results suggest that REIC/Dkk-3 has a potential role in canine androgen-independent prostate cancer as a novel anti-cancer agent.

Moreover, SGTA regulates the cell cycle, protein folding, transcription, protein transport, ubiquitin-proteasomes, and hormone receptor signalling [17, 31]. Co-chaperone proteins, including SGTA, interact with chaperones such as Hsp90 [18]. Hsp90 and other chaperone proteins are overexpressed in cancer cells with co-chaperones [18]. These events suggest that the expression of SGTA is induced by the overexpression of chaperones in various cancers types, not only prostate cancer [e.g.*,* oesophageal squamous cell carcinoma and breast carcinoma] [31, 32]. Although upregulation of SGTA expression may not be related to hormonal signalling, overexpressed SGTA plays an important role as an inhibitor of AR signalling and confers the androgen-independent characteristics in prostate cancer [3]. In our previous study, canine SGTA was not overexpressed in prostate hyperplasia in both castrated and not castrated dogs [20]. However, castrated dogs with prostatic carcinoma highly overexpressed SGTA [20]. These results suggest that an excess of AR signalling is not a trigger for SGTA overexpression in the stage between hyperplasia and prostatic cancer in dogs, but sequentially SGTA overexpression induces the inhibition of AR signalling which leads to androgen-independency. This characteristic is remarkably similar to human androgen-independent prostate cancer. Therefore, the investigation of the relationship between AR signalling and SGTA function in canine prostate cancer allows a better understanding of the mechanisms of formation of androgen-independent prostate cancer beyond the species.

Recently, we showed that SGTA is expressed in canine prostate cancer but not in prostate hyperplasia [20]. The CHP-1 cell line, which was established from a sample of canine prostate cancer, was positive for cytokeratin AE1/AE3, an epithelial marker [5]. Although prostate cancer CHP-1 cells, which were obtained from a castrated dog, do not express the AR, the cells with AR exhibited androgen-dependent AR signalling (Fig. 3). These results suggest that CHP-1 have acquired androgen-independency followed by cancer progression. Therefore, CHP-1 may have similar characteristics to human androgen-independent prostate cancer cells, e.g. PC3 [5]. We demonstrated that REIC/Dkk-3 was not only an anti-cancer agent that induces tumour specific apoptosis but also restores AR signalling in both CHP-1 and human androgen-independent prostate cancer PC3 cells [19]. Compared to humans, castration is a very common procedure for preventing bad habits and male-specific diseases in dogs. Our results suggest that castrated dogs, and derived cells and tissues, are useful models of the study of both human and canine end-stage androgen-independent prostate cancers. The REIC/Dkk-3 and SGTA proteins are structurally and functionally well-conserved between human and dog [20, 30]; thus, our results regarding the interaction of these molecules in androgen-independent prostate cancer of human and dogs are applicable to both species. Further analysis regarding the mechanisms of the AR complex maturation via REIC/Dkk-3 and SGTA interaction in in vitro and in vivo experiments should contribute to the development of a novel strategy for the treatment of canine androgen-independent prostate cancer.

## Conclusions

In conclusion, canine REIC/Dkk-3 interacts with SGTA and this interaction rescues AR signalling in both human and canine androgen-independent prostate cancers. Moreover, these results show that the role of canine REIC/Dkk-3 in AR signalling recovery can be replicated in canine prostate cancer cells. Further analyses regarding the role of REIC/Dkk-3 and SGTA interaction should contribute to breaking through refractory canine androgen-independent prostate cancer.

## Additional file

Additional file 1: Table S1. Primer sets used in this study. (XLSX 9 kb)

## Abbreviations

AR: Androgen receptor
DHT: Dihydrotestosterone
ER: Endoplasmic reticulum
FBS: Foetal bovine serum
HA: Haemagglutinin
JNK: c-Jun-NH2-terminal kinase
MTH: Mammalian two-hybrid
ORF: Open reading frame
PD: Pull-down
PSA: Prostate specific antigen
REIC/Dkk-3: Reduced expression in immortalised cells
SGTA: Small glutamine-rich tetratricopeptide repeat-containing protein α
